# Longitudinal changes in task-evoked brain responses in Parkinson's disease patients with and without mild cognitive impairment

**DOI:** 10.3389/fnins.2014.00207

**Published:** 2014-07-29

**Authors:** Urban Ekman, Johan Eriksson, Lars Forsgren, Magdalena E. Domellöf, Eva Elgh, Anders Lundquist, Lars Nyberg

**Affiliations:** ^1^Department of Integrative Medical Biology, Umeå UniversityUmeå, Sweden; ^2^Department of Radiation Sciences, Diagnostic Radiology, Umeå UniversityUmeå, Sweden; ^3^Department of Pharmacology and Clinical Neuroscience, Umeå UniversityUmeå, Sweden; ^4^Umeå Center for Functional Brain Imaging, Umeå UniversityUmeå, Sweden; ^5^Department of Clinical sciences, Psychiatry, Umeå UniversityUmeå, Sweden; ^6^Department of Statistics, USBE, Umeå UniversityUmeå, Sweden

**Keywords:** Parkinson disease, MCI, longitudinal, working memory, functional MRI (fMRI)

## Abstract

Cognitive deficits are common in Parkinson's disease. Previous cross-sectional research has demonstrated a link between cognitive impairments and fronto-striatal dopaminergic dysmodulation. However, longitudinal studies that link disease progression with altered task-evoked brain activity are lacking. Therefore, our objective was to longitudinally evaluate working-memory related brain activity changes in Parkinson's disease patients with and without mild cognitive impairment (MCI). Patients were recruited within a longitudinal cohort study of incident patients with idiopathic parkinsonism. We longitudinally (at baseline examination and at 12-months follow-up) compared 28 patients with Parkinson's disease without MCI with 11 patients with Parkinson's disease and MCI. Functional MRI blood oxygen level dependent signal was measured during a verbal two-back working-memory task. Patients with MCI under-recruited bilateral medial prefrontal cortex at both time-points (main effect of group: *p* < 0.001, uncorrected). Critically, a significant group-by-time interaction effect (*p* < 0.001, uncorrected) was found in the right fusiform gyrus, indicating that working-memory related activity decreased for patients with Parkinson's disease and MCI between baseline and follow-up, while patients without MCI were stable across time-points. The functional connectivity between right fusiform gyrus and bilateral caudate nucleus was stronger for patients without MCI relative to patients with MCI. Our findings support the view that deficits in working-memory updating are related to persistent fronto-striatal under-recruitments in patients with early phase Parkinson's disease and MCI. The longitudinal evolution of MCI in Parkinson's disease translates into additional task-evoked posterior cortical changes.

## Introduction

Cognitive impairment frequently accompanies the characteristic motor deficits in Parkinson's disease (PD), ranging from mild cognitive impairment (MCI) to dementia (Kehagia et al., 2010; Svenningsson et al., 2012). Approximately 20–40% of patients with PD have MCI at an early phase (Aarsland et al., 2010), and the risk of developing PD dementia (PDD) is markedly increased for patients with MCI compared to patients without MCI (Janvin et al., 2006; Broeders et al., 2013; Pedersen et al., 2013).

Previous cross-sectional research has linked cognitive impairments in PD to both structural and functional brain deficits (Kehagia et al., 2010; Svenningsson et al., 2012), and early-phase alterations are commonly related to working-memory and executive processes (Owen, 2004; Monchi et al., 2007; Marklund et al., 2009). In a previous cross-sectional study, we showed that patients with PD and MCI under-recruited bilateral anterior cingulate cortex (ACC) and right caudate nucleus compared with patients without MCI during working-memory updating (Ekman et al., 2012). Additionally, a recent publication reported findings in accordance with our study (Nagano-Saito et al., 2014). Fronto-striatal hypo-activity has also been demonstrated for patients with PD and impaired executive functions relative to non-impaired patients (Lewis et al., 2003), and impaired working-memory is associated with nigro-striatal (Rinne et al., 2000; Cools, 2006; Ekman et al., 2012), and meso-cortical (Mattay et al., 2002; Monchi et al., 2007) dopaminergic dysfunction.

Cross-sectional studies have thus provided information on the anatomical and neurochemical bases of cognitive deficits in PD. However, longitudinal cohort studies are fundamental in order to better understand underlying mechanisms (Kehagia et al., 2010; Monchi and Stoessl, 2012). Research using longitudinal methodology is generally more sensitive in detecting brain changes, and not as susceptible to cohort differences as cross-sectional methodology (Raz, 2005; Nyberg et al., 2010). However, only a limited number of longitudinal studies have linked brain changes to cognitive decline in PD. Functional changes have been related to evolving glucose metabolism decline within cognitive networks of prefrontal- and parietal cortices in patients with PD (Huang et al., 2007), and tests probing posterior cortical function rather than tests probing fronto-striatal cortical function have been demonstrated to enhance predictions of global cognitive decline (Williams-Gray et al., 2007, 2013). Critically, no longitudinal study has previously evaluated task-evoked brain responses in relation to cognitive impairments in PD. Task-evoked methodology offers a unique possibility to evaluate physiological responses within the operative brain in relation to cognitive deficits. Thus, the aim of this study was to longitudinally (at time for initial PD-diagnoses and at 12-months follow-up) assess changes in working-memory related brain responses using functional MRI in a population-based cohort of patients with PD, with and without MCI. Because patients with PD and MCI have increased risk for PDD (Janvin et al., 2006; Broeders et al., 2013), we expect that a large proportion of the patients with MCI eventually progress to PDD. However, although a large proportion of patients with MCI convert to dementia, some patients never progress or even revert to normal cognition, which implies heterogeneous causes (Richard and Brayne, 2014). To ensure that the patients with PD and MCI did not convert to normal cognition or PDD across time (i.e., to increase the prognostic accuracy of MCI in respect to prodromal PDD), repeated neuropsychological testing was carried out (Pedersen et al., 2013). Global cognitive decline might be related to neural dysfunction with posterior cortical basis rather than fronto-striatal circuits (Huang et al., 2007; Williams-Gray et al., 2007). Therefore, we hypothesized that posterior cortical changes across time might be more pronounced than fronto-striatal changes in patients with PD and MCI, reflecting progression toward prodromal PDD.

## Materials and methods

### Participants

The recruitment process was conducted within the “newly diagnosed parkinsonism in Umeå” (NYPUM) project, which is a longitudinal population-based cohort study of incident patients with idiopathic parkinsonism, including PD. All physicians in the Umeå catchment area (about 142 000 inhabitants) were continually requested for referral of all patients with suspected parkinsonism to the Department of Neurology at Umeå University, during the inclusion interval January 1, 2004, to April 30, 2009. Patients were included if they fulfilled the UK Parkinson's Disease Society Brain Bank clinical diagnostic criteria for definite PD (UKPDS; Gibb and Lees, 1988) at the latest available re-evaluation (36–60 months after initial diagnosis). The participants performed the first (baseline) neuropsychological examination and fMRI scanning approximately 1–2 months after initial PD-diagnosis, and all participants were drug-naïve regarding dopaminergic medication at that time-point (see Table 1 for demographic data). However, at the time for the first follow-up (Mean = 12 months and 23 days, *SD* = 2 months and 15 days) all participants (except one cognitively healthy participant) received dopaminergic anti-parkinsonian medication on a daily basis. Dopamine treatment were recorded and calculated as levodopa equivalent dose (LED) according to the conversion factors used by Tomlinson et al. (2010). This study was approved by the ethics committee of the Faculty of Medicine at Umeå University, Umeå, Sweden.

**Table 1:** **Demographics and clinical characteristics**.

	**PDMCI^+^ (*n* = 11)**		**PDMCI^−^ (*n* = 28)**		**Difference at baseline**	**Difference at 12-months follow-up**	**Main effect of group**	**Group-by-time interaction**
**Male/female (bimanual *p*-value)**	9/2 (*p* = 0.07)		13/15 (*p* = 0.85)		n.a.	n.a.	n.a.	n.a.
**Years of education**	9.55 (3.9)		12.5 (5.4)		0.10	n.a.	n.a.	n.a.
**Age at baseline examination**	65.9 (9.0)		67.5 (10.2)		0.66	n.a.	n.a.	n.a.
**Years between symptom onset and PD-diagnosis**	2.22 (2.2)		2.0 (1.5)		0.76	n.a.	n.a.	n.a.
**Time-points**	Baseline	12-months	Baseline	12-months				
**Mine-mental state examination**	28.8 (1.3)	28.7 (1.6)	29.4 (0.8)	29.4 (0.9)	0.12	0.11	0.47	0.77
**UPDRS-III motor scores**	31.5 (12.0)	23.2 (12.2)	22.9 (9.2)	20.3 (9.2)	0.01	0.21	0.91	0.47
**LED**	0	422.7 (84.0)	0	296.4 (159.7)	n.a.	0.01	n.a.	n.a.
**MADRS**	4.3 (3.7)	2.9 (2.1)	4.4 (3.4)	3.6 (3.7)	0.41	0.26	0.71	0.69
**Executive functions**	41.9 (5.9)	42.2 (15.1)	54.1 (10.5)	59.7 (14.1)	<0.01	<0.01	<0.01	0.13
**Episodic memory**	37.5 (8.4)	37.7 (7.2)	514 (5.3)	52.2 (8.3)	<0.01	<0.01	<0.01	0.95
**Attention/working memory**	33.9 (8.1)	36.2 (7.0)	47.4 (5.5)	47.3 (5, 1)	<0.01	<0.01	<0.01	0.21
**Language**	51.6 (12.5)	No values	52.3 (13.4)	No values	0.44	n.a.	n.a.	n.a.
**Visuospatial functions**	50.1 (9.2)	52.2 (4.6)	57.4 (3.4)	56.6 (5.2)	<0.01	0.02	<0.01	0.14

The data are mean values with standard deviations in parentheses. The five cognitive domains for assessing MCI (executive functions, episodic memory, attention/working-memory, language and visuospatial functions) represent means of the included domain-specific tests age-matched t-values. n, numbers of participants. UPDRS-III (unified Parkinson's disease rating scale, part 3) and LED (levodopa equivalent doses). MADRS (Montgomery and Åsberg Depression Rating scale). n.a., not applicable. Analyses of group differences at baseline, 12-months, main effect of group, and group-by-time interaction are reported as p-values.

### Classification of MCI and final groups

**Figure 1:**
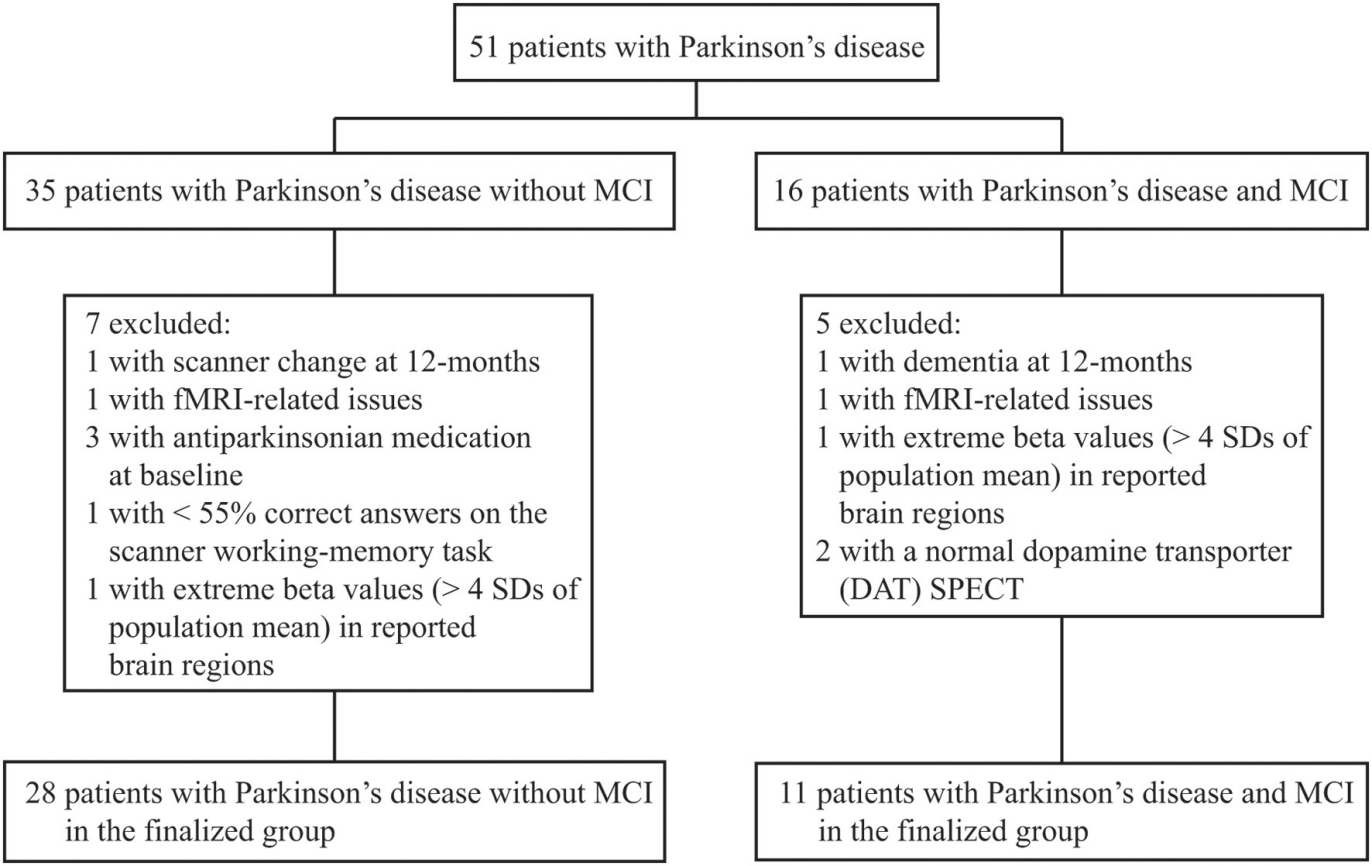
**Flow-chart of study profile.** Of 67 enrolled Parkinson's disease patients, 51 had conducted neuropsychological assessments and fMRI at both time-points with either a manifested MCI or were cognitive stable. SD, standard deviation.

We classified MCI by assessing The Movement Disorders Society commissioned taskforce criteria for MCI in PD (Litvan et al., 2012) in the same fashion as previously described (Ekman et al., 2012). In brief, the included neuropsychological tests tapped five different cognitive domains: executive functions [Wisconsin Card Sorting Test (WCST) (total errors and perseverative responses) and animal fluency], attention/working-memory (digit span backwards and trail making test, part B), episodic memory [Brief Visuospatial Memory Test revised, (average scores of free and delayed recall); Free and Cued Selective Reminding Test, free recall], visuospatial function (Judgment of Line Orientation Test), and language [Boston Naming Test (BNT)]. Patients who scored ≥1.5 *SD*s below the normative age-matched mean value in at least two cognitive test measures were classified with MCI. Because only one cognitive measure was assessed in the language and visuospatial domains, MCI was classified on level I criteria (i.e., subtyping not possible). Because we had available neuropsychological data at 36-monts follow-up, we controlled that results at 36-months follow-up confirmed the MCI classifications set at 12-months to increase accuracy. In conformity with a recent large-scale pooled study (Aarsland et al., 2010), no subjective measures were part of the MCI classification. However, all but one participant classified with MCI reported complaints of subjective cognitive decline. The exclusion process is described in Figure 1. Dementia was assessed using consensus criteria (Emre et al., 2007), reflecting both subjective (deficits severe enough to significantly impair daily life independent of impairment due to non-cognitive symptoms of PD assessed with a clinical interview and the Parkinson's disease questionnaire 39) and objective measures (cognitive impairment in at least two cognitive domains with performance ≥2 *SD*s below normative age-matched *t*-values; one patient was excluded with suspected dementia of Lewy-Body type). The cut-off for major depression was set to a score of >17 (according to the Montgomery and Åsberg Depression Rating scale; Montgomery and Åsberg, 1979), but no participants were excluded due to depression. Patients classified with MCI at baseline but not at 12-, or 36-months follow-up were excluded due to unclear cognitive status. After the initial exclusions, the sample consisted of 39 participants whom all conducted the neuropsychological- and fMRI examinations at both time-points with satisfactory data collection (one participant lacked neuropsychological data at 12-months, but was confirmed as MCI at 36-months). The final groups consisted of 11 participants that had PD with MCI (PDMCI^+^) at both time-points, and 28 participants who had PD without MCI (PDMCI^−^) at both time-points. Out of the 11 PDMCI^+^ participants, seven were treated with levodopa at 12-months follow-up, three with a combination of levodopa and dopamine agonists, and the last patient with dopamine agonists singly. The equivalent number for PDMCI^−^ were: 12 were treated with dopamine agonists, 10 with levodopa, five with a combination of levodopa and dopamine agonists, and the last patient did not receive any dopaminergic medication at 12-months follow-up.

### Experimental procedures and fMRI data acquisition

The procedures for the fMRI data acquisition have been described previously (Ekman et al., 2012). In brief, during scanning participants performed a verbal working-memory 2-back updating task. The task demanded the participants to actively maintain and update information (nouns) that regularly were presented on the screen. The participants were instructed to respond “yes” (right index finger) when the presented noun matched the one two items earlier and “no” (left index finger) when it differed, by pushing MRI-compatible keypads. During the baseline condition participants were instructed to do nothing except keeping their gaze fixed on a small circle that were displayed at the center of the screen. The fMRI acquisition was conducted using two different scanners: a 1.5T Philips Intera scanner and a 3T Philips Achieva scanner (both scanners from Philips, Eindhoven, Netherlands). The sequences had the following parameters for the 1.5T scanner: repetition time (TR): 3000 ms, echo time (TE) 50 ms, flip angle 90°, field of view: 22 × 22 cm, 64 × 64 matrix and 4.40 mm slice thickness (voxel size 3.44 × 3.44 × 4.40 mm). During every repetition time, 33 slices were acquired. To avoid effects of signal saturations, five dummy scans were performed prior to the image acquisition. Acquisition parameters for the 3T scanner were: TR 1500 ms, TE 30 ms, flip angle 70°, field of view 22 × 22, 64 × 64 matrix and 4.65 mm slice thickness (voxel size 3.44 × 3.44 × 4.65). During every repetition time, 31 slices were acquired. Ten dummy scans were performed prior to the image acquisition. For PDMCI^−^ the scanner distributions were: 19 participants performed the working memory task in the 1.5T scanner and 9 participants in the 3T scanner. The equivalent number for PDMCI^+^ were 6 in the 1.5T scanner and 5 in the 3T scanner. All included participants conducted the experiment on the same scanner at both time-points.

### Statistical analyses

Preprocessing and statistical analyses of fMRI-data were done with Statistical Parametric Mapping version 8 (SPM 8, Welcome Department of Imaging Neuroscience, London, UK) run in Matlab (MathWorks). The preprocessing included slice-timing correction within each volume to adjust for differences in slice acquisition, Movement correction were performed by realign and unwarp to the first image in the series. To consider group-specific anatomical brain differences, all participants were initially normalized to the Montreal Neurological Institute (MNI) echoplanar-imaging template. In a second step, normalization was done to group-specific means for patients with and without MCI at each time point. The mean-normalized brain templates were then projected to the total mean of all participants. Finally, the images were smoothed by using an 8 mm FWHM isotropic Gaussian filter kernel. For functional MRI data, effects were modeled in the framework of the general linear model (GLM; Friston et al., 1995). The event-related fMRI responses were modeled as regressors containing delta functions that represented onsets of word stimuli, and the regressors were convolved with a canonical haemodynamic response function. Model estimations from each participant were input into a second-level factorial analysis with the factors group, time-point, subject, and scanner. Motor scores (UPDRS-III) and levodopa equivalent doses (LED; daily levodopa and dopamine agonist dosages combined) for each participant were input as covariate in the model due to significant group differences (Table 1). Due to the low number of patients treated with dopamine agonists in PDMCI^+^, no sub-analyses of potential medication effects on cognition were conducted. The statistical threshold was set to *P* < 0.001, uncorrected, and the cut-off for number of contiguous voxels (k) was ≥10 in the whole-brain fMRI analyses. Results significant at *p* < 0.005 (≥ 10 contiguous voxels) are reported as a trend.

To evaluate possible confounding effects from scanner differences, we performed *F*-tests (*P* < 0.001) on main effect of scanner, group-by-scanner, time-by-scanner, and group-by-time-by-scanner interactions (Stonnington et al., 2008). Pooled analyses have previously been conducted to handle MRI-scanners with different magnetic field strengths (Abdulkadir et al., 2011; Ekman et al., 2012; Marchewka et al., 2014). Furthermore, we analyzed effect sizes (Cohen's *d*) on each scanner separately in our reported main effects to ensure equivalence across scanners. One region-of-interest (ROI) analysis in the right caudate was conducted, and the ROI has been previously defined (Ekman et al., 2012).

A psychophysiological interaction (PPI) analysis was performed to evaluate how a ROI functionally interacted with other brain areas during task-performances (Friston et al., 1997). In brief, we performed a second GLM analysis that included one regressor representing the BOLD signal time course in a given ROI, one regressor representing the psychological variable of interest (i.e., working memory condition>baseline condition), and finally one regressor representing the interaction of the former two. The ROIs signal time course (contrast vectors: task condition>baseline condition) for each individual was extracted from a sphere (radius of 6 mm) centered on the seeds peak voxel (derived from the group-by-time interaction post-hoc analysis). The statistical threshold in the PPI-analysis was set to *p* < 0.001, and the cut-off for number of contiguous voxels was ≥10.

Longitudinal analyses of the right caudate ROI, demographics, clinical characteristics, and the scanner-task behavioral data were assessed within the framework of the GLM using a two-factor (group-by-time) repeated-measures ANOVA. Analyses of group differences at a specific time-point were conducted using independent-samples *t*-test. The statistical threshold in the ROI- and the behavioral analyses was set to *p* < 0.05. Group comparisons of behavioral performances were analyzed as one-tailed due to á priori hypotheses, and demographic data were analyzed as two-tailed. Gender distribution was analyzed with a binominal test.

## Results

### Behavioral data

Neuropsychological testing revealed that, PDMCI^+^ had significantly reduced performances (main effect of group across baseline and 12-months) within all cognitive domains (except the language domain that was assessed only at baseline) compared to PDMCI^−^ (Table 1). There were no significant group-by-time interaction effects or main effects of time.

**Figure 2:**
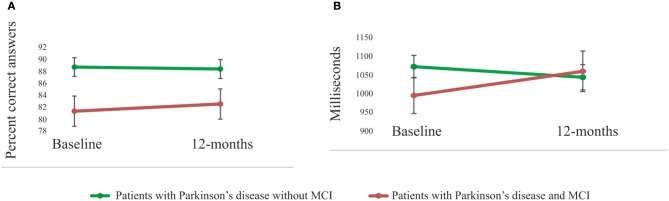
**Performances on the scanner working-memory task. (A)** A significant (*p* = 0.01) main effect of group in performance accuracy was shown with superior performances for patients with Parkinson's disease without MCI compared to patients with MCI. The group-by-time interaction effect was not significant (*p* = 0.65). **(B)** There was no significant group difference in reaction time (main effect, *p* = 0.58), but the group-by-time interaction effect was close to significant (*p* = 0.07).

As shown in Figure 2, a significant main effect of group was also evident when comparing accuracy (percentage correct answers) on the scanner working-memory 2-back task [*F*_(1, 36)_ = 7.12, *p* = 0.01]. There was no main effect of group regarding reaction times [RT; *F*_(1, 36)_ = 0.31, *p* = 0.58]. The group-by-time interaction effect was not significant for performance accuracy [*F*_(1, 36)_ = 0.21, *p* = 0.65], but approached significance for RTs [*F*_(1, 36)_ = 3.43, *p* = 0.07]. There was no main effect of time (*p* > 0.10).

### Neuroimaging data

#### Whole-brain analyses

**Figure 3:**
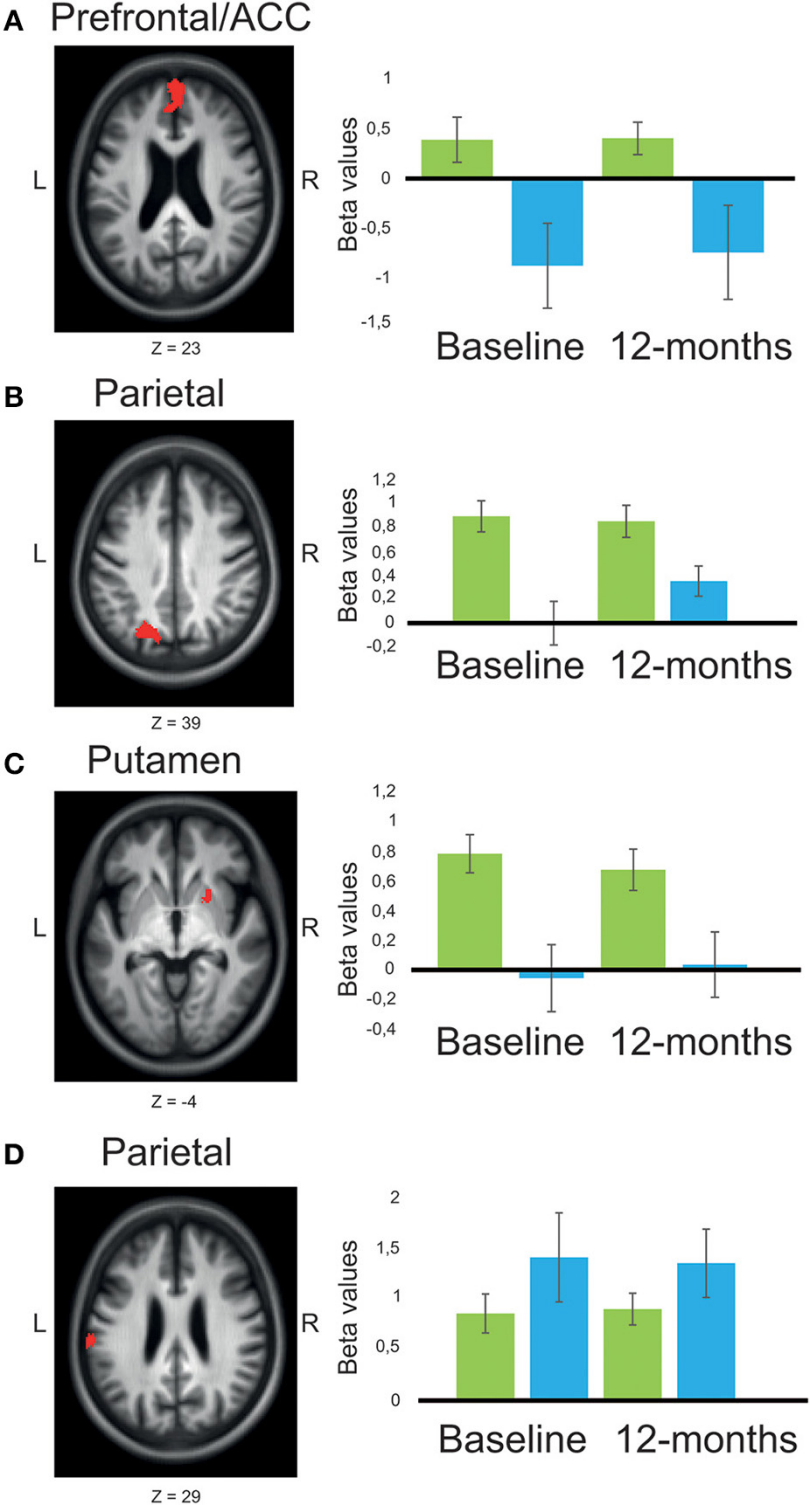
**Main effects of group across time-points, comparing BOLD-signal intensity in patients with Parkinson's disease with or without MCI. (A)** The presented results are chosen to illustrate fronto-striatal respective posterior cortical circuitry (with two opposite patterns). significant main effect of group (red) was shown in bilateral medial PFC/ACC **(A)**, left superior parietal/occipital cortex **(B)**, and putamen **(C)**, where patients with MCI (*n* = 11) showed reduced BOLD-signal intensity compared to patients without MCI (*n* = 28). An opposite pattern was shown in left supramarginal gyrus **(D)** were patients with MCI showed larger BOLD-signal intensity compared to patients without MCI. Mean beta values are presented as plots contrasting working-memory 2-back task with the baseline resting condition for patients with Parkinson's disease without MCI (green), and patients with MCI (blue). Error bars are 1 SE. Z, anatomical location in Montreal Neurological Institute transversal space. The statistical threshold in the pictures (red clusters) is set to *p* < 0.005 to increase visibility.

When comparing working-memory related BOLD-signal intensity, PDMCI^+^ displayed significant under-recruitment compared to PDMCI^−^. This was seen as a main effect of group in bilateral medial prefrontal cortex, left precentral gyrus, and as a trend in left orbitofrontal cortex, left superior frontal gyrus, right postcentral gyrus, and in the right inferior temporal cortex (Table 2, Figure 3). No overlap was observed between our reported main effects of group and a main effect of scanner analysis, or a group-by-scanner analysis. Furthermore, quantifications of the responses for the two scanners separately showed consistent magnitude of BOLD-signal change on both scanners, with large effect sizes (Cohen's *d* > 0.80). Less conclusive evidence due to potential influences of the scanner factor was observed in right putamen, left parietal cortex, left cuneus, precuneus, and the left occipital cortex (Supplementary Table 1).

**Table 2:** **Main effects of group in BOLD-signal intensity between patients with Parkinson's disease with and without MCI**.

**Brain region**	**Side**	**Peak (*x, y, z*)**	***F***	***K***
**PATIENTS WITHOUT MCI > PATIENTS WITH MCI**				
**Frontal**				
Precentral gyrus	L	−42, 4, 30	19.29	23
Medial prefrontal	L/R	0, 54, 28	22.12	148
Orbitofrontal cortex^*^	L	−24, 34, −12	15.92	42
Superior frontal gyrus^*^	L	−22, 16, 64	14.93	24
**Parietal**				
Postcentral gyrus^*^	R	44, −18, 48	14.87	48
**Temporal**				
Inferior temporal^*^	R	48, −18, −22	14.42	39
**PATIENTS WITHOUT MCI < PATIENTS WITH MCI**				
**Frontal**				
Orbitofrontal cortex	L	−8, 60, −8	17.28	13
**Parietal**				
Supramarginal	L	−64, −24, 30	17.22	13

The coordinates x, y, z refers to the anatomical location of the Montreal Neurological Institute space for the clusters local maxima. p < 0.001;

* p < 0.005. L, left; R, right; F, F-values; k, number of voxels.

**Figure 4:**
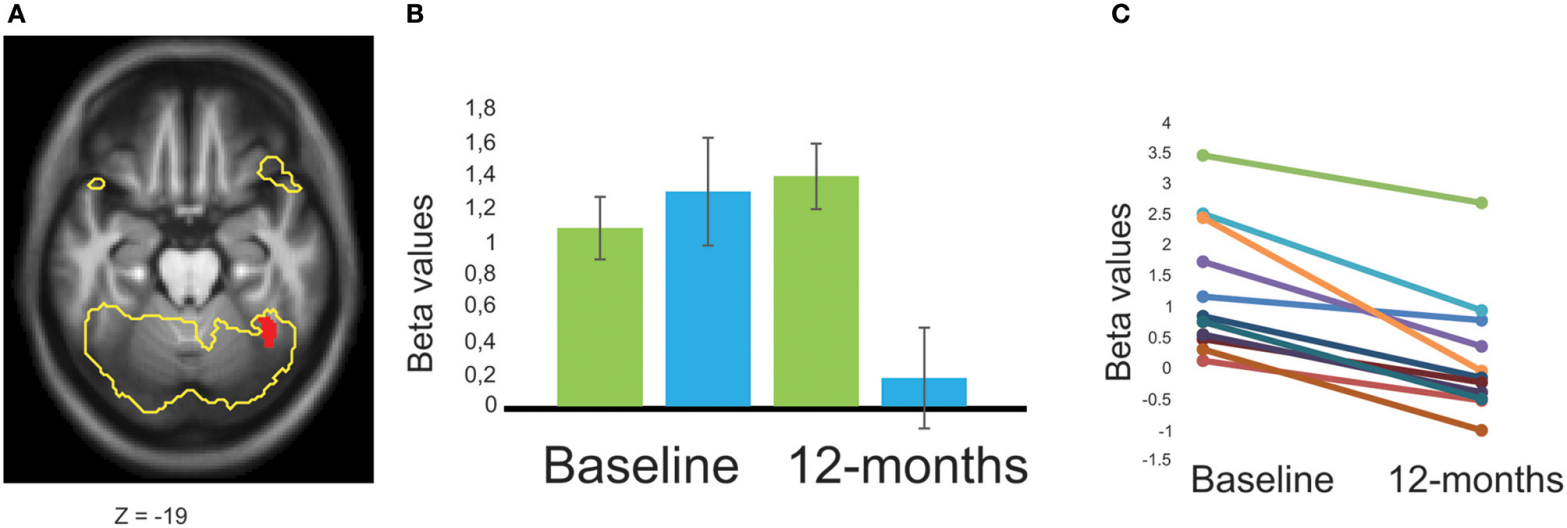
**Group-by-time interaction effect, comparing BOLD-signal intensity in patients with Parkinson's disease with or without MCI. (A)** A significant group-by-time interaction effect (red) was shown in the right fusiform gyrus comparing patients with Parkinson's disease and MCI (*n* = 11) to patients without MCI (*n* = 28). The task-specific activation for all included participants with Parkinson's disease are presented as a yellow outline to illustrate the contrast between 2-back working-memory processing and resting baseline-condition (*p* < 0.05, family-wise error corrected). **(B)** Mean beta values are presented as plots for patients with Parkinson's disease without MCI (green), and with MCI (blue) at both time-points. **(C)** All patients with Parkinson's disease and MCI showed a negative slope across time with decreased BOLD-signal intensity (each participants is represented as a line in the figure). Error bars are 1 SE. Z, anatomical location in Montreal Neurological Institute transversal space.

A group-by-time interaction effect was evident in the right fusiform gyrus (Figure 4), right vermis, and as a trend in left inferior temporal cortex (Table 3). Specifically, PDMCI^+^ decreased their BOLD-signal intensity in the fusiform gyrus between baseline and 12-months, while PDMCI^−^ were stable across both time-points. By contrast, PDMCI^+^ increased their BOLD-signal intensity between baseline and 12-months in right parietal cortex and left prefrontal cortex while PDMCI^−^ were again stable across time-points. No overlap was observed between our reported group-by-time interaction effects, and a scanner-by-time interaction, a group-by-scanner interaction, or a group-by-time-by-scanner interaction effect. Less conclusive evidence due to potential influences of the scanner factor, was observed in right cerebellum and in left dorsolateral prefrontal cortex (Supplementary Note 1).

**Table 3:** **Group-by-time interaction effects in BOLD-signal intensity between patients with Parkinson's disease with and without MCI**.

**Brain regions**	**Side**	**Peak (*x, y, z*)**	***F***	***K***
**PATIENTS WITH MCI SHOW DECREASED BOLD-SIGNAL INTENSITY LONGITUDINALLY**				
Fusiform gyrus	R	38, −48, −22	26.45	86
Vermis	R	4, −52, −20	19.87	19
Inferior temporal^*^	L	−48, −2, −38	13.25	23
**PATIENTS WITH MCI SHOW INCREASED BOLD-SIGNAL INTENSITY LONGITUDINALLY**				
Dorsolateral prefrontal^*^	L	−42, 44, 26	14.43	45
Superior parietal^*^	R	22, −52, 60	13.36	26

The coordinates x, y, z refers to the anatomical location of the Montreal Neurological Institute space for the clusters local maxima. p < 0.001;

* p < 0.005. L, left; R, right; F, F-values; k, number of voxels.

We performed a PPI-analysis to evaluate if the right fusiform gyrus had functional connectivity with additional working-memory related brain regions. A main effect of group revealed that PDMCI^−^ had stronger functional connectivity relative to PDMCI^+^ between right fusiform gyrus and bilateral caudate nucleus (15 voxels located in MNI-space *x* = −16, *y* = 18, *z* = 4, and 13 voxels in *x* = 18, *y* = 10, *z* = 14), and as a trend in right pre-central gyrus (32 voxels in *x* = 56, *y* = 8, *z* = 40), Less conclusive evidence due to potential influences of the scanner factor, was observed in left pre-central gyrus, and in left superior intraparietal sulcus (Supplementary Note 2). The functional connectivity between right fusiform gyrus and other brain regions did not decline as function of time.

#### ROI analysis

In a previous study (Ekman et al., 2012), we reported that PDMCI^+^ under-recruited parts of the ACC and also the right caudate already at the time for initial PD-diagnosis compared to PDMCI^−^. In keeping with those findings, the group differences remained in parts of the ACC and contiguous areas as shown in the whole-brain analyses of our current study. However, the whole-brain analysis did not reveal any group difference in the caudate, and we therefore applied the right caudate ROI from our previous cross-sectional study (Ekman et al., 2012). The group difference in the right caudate ROI was weakened at follow-up and the main effect of group was now only approaching significance (*p* = 0.07).

## Discussion

In a population-based cohort of newly diagnosed patients with incident PD, we showed that PDMCI^+^ performed inferior across time-points on the fMRI working-memory task compared to PDMCI^−^. Evaluations of longitudinal changes in functional brain responses showed a group-by-time interaction effect in the right fusiform gyrus, where PDMCI^+^ longitudinally decreased their BOLD-signal intensity whereas PDMCI^−^ were stable across time-points. In addition to the longitudinal change, compared to PDMCI^−^, PDMCI^+^ displayed under-recruited BOLD-signal intensity across time in medial prefrontal cortex.

The fusiform gyrus is primarily associated with higher-order visual processing, but also with visual working-memory processing (Cuortney et al., 1997; Wager and Smith, 2003; Chang et al., 2007; Rottschy et al., 2012). It has been hypothesized that working-memory maintenance may involve the same cortical regions that initially process the representations to be held in working-memory (D'Esposito, 2007). We confirmed with the PPI analysis that the right fusiform gyrus is functionally connected with bilateral caudate nucleus, bilateral pre-central gyrus and left intraparietal sulcus during working-memory processing (Gazzaley et al., 2004). Critically, such connectivity was more robust for PDMCI^−^ compared to PDMCI^+^. The reported alterations in the right fusiform gyrus may thus reflect a longitudinal change in PDMCI^+^ that is related to working-memory maintenance. However, the right fusiform gyrus change might precede additional cognitive decline for PDMCI^+^ at follow-up (Bateman et al., 2012). Structural changes in the fusiform gyrus have been reported for patients with PD and MCI compared to patients without MCI (Pagonabarraga et al., 2013), as well as marked reductions in gray matter density in patients with PDD (Nagano-Saito et al., 2005; Ramírez-Ruiz et al., 2005). Functional changes have been related to evolving glucose metabolism decline within cognitive networks with both posterior and prefrontal cortical basis in patients with PD (Huang et al., 2007), but impairments in cognitive measures mediated by pathology in posterior cortical regions rather than fronto-striatal regions have been proposed to predict prodromal PDD (Williams-Gray et al., 2007, 2013). Correspondences of brain atrophy in temporal-parietal regions between patients with PD and Alzheimer's disease have also been reported to predict long-term cognitive decline (Weintraub et al., 2012).

In a previous cross-sectional study, we reported that newly diagnoses drug-naïve patients with PDMCI^+^ under-recruited bilateral ACC and right caudate during working-memory updating (Ekman et al., 2012). In agreement with that, we show in the present study that PDMCI^+^ under-recruited bilateral medial prefrontal cortex (including ACC) compared to PDMCI^−^, and that the alterations persist across time. ACC is related to high-level cognitive processing (Duncan and Owen, 2000), and PD-related alterations are associated with striatal dopaminergic depletions (Ito et al., 2002), decreased metabolism (Polito et al., 2012), and loss of neuronal integrity (Lewis et al., 2012).

In agreement with our previous cross-sectional study, persistent striatal alterations were shown in the present study, where PDMCI^+^ showed inferior BOLD-signal intensity in right putamen and in the right caudate ROI compared to PDMCI^−^. However, a main effect of scanner overlapped with the main effect of group in the right putamen, which make this evidence less conclusive, even though large scanner-specific effect sizes were evident. Striatal alterations are repeatedly reported in the early phase of PD in relation to cognitive impairment and depleted dopaminergic circuitry (Sawamoto et al., 2008; Kehagia et al., 2010; Ekman et al., 2012), and dopamine synthesis capacity in the putamen and caudate positively correlates with working-memory capacity (Cools et al., 2008). A weakened group difference was revealed in the right caudate ROI when comparing PDMCI^+^ to PDMCI^−^ longitudinally, potentially reflecting a beneficial medication effect due to onset of anti-parkinsonian treatment. A positive treatment effect might also partly explain the lack of further behavioral decline for PDMCI^+^ on the scanner working-memory task, and on the cognitive tests outside the scanner. However, anti-parkinsonian dopaminergic medication has previously shown both positive and negative outcomes on cognitive functions (Cools, 2006), and given the relatively small sample size, potential cognitive effects of dopaminergic medication on brain activation is speculative.

The reported alterations might be associated to additional neurochemical dysfunction. Hypo-metabolism have been reported in patients with PD and multi-domain MCI compared to patients with normal cognition in lateral frontal cortex, ACC, and parietal-temporal-occipital cortices (Lyoo et al., 2010). Patients with PD commonly demonstrate cortical cholinergic dysfunction that is more pronounced in PDD (Bohnen et al., 2003), and covariance between striatal ^18^F-fluorodopa (FDOPA) reduction and decreased cortical cholinergic binding in frontal and temporal-parietal cortices have been reported in PDD (Hilker et al., 2005). Furthermore, degeneration of locus coeruleus (LC) might affect executive processes due to deficits in norepinephrine (NE) transmission (Marsh et al., 2009; Vazey and Aston-Jones, 2012). The LC region mainly projects to parietal, temporal, and frontal regions. Thus, the current findings suggest an early evolving pattern in PDMCI^+^ consisting of posterior cortical change that might relate to evolving norepinephrine and/or cholinergic dysfunction.

In contrast to inferior BOLD-signal intensity for PDMCI^+^, we report group-by-time interaction effects in right parietal cortex indicating up-regulated BOLD-signal intensity between baseline and follow-up for PDMCI^+^, whereas PDMCI^−^ were stable across time. Additionally, PDMCI^+^ recruited parts of the left supramarginal gyrus to a larger extent than PDMCI^−^ across time. The supramarginal gyrus has been proposed as part of the phonological loop and is associated to working-memory span and interference control (Burgess et al., 2011). The reported up-regulations could potentially reflect increased effort in performing the fMRI working-memory task due to evolving cognitive impairments related to fronto-striatal and temporal circuitry. The increased activation might also relate to compensatory regulations for the observed fronto-striatal alterations, and might in part explain the lack of additional behavioral decline at follow-up.

The longitudinal method is a strength as it increases the ability to generalize the reported effects (Raz, 2005; Nyberg et al., 2010), whereas the repeated neuropsychological assessments increases the prognostic accuracy of MCI with respect to prodromal PDD (Pedersen et al., 2013). Data acquisition was conducted on two different scanners and is a limitation that might have induced additional variability. The detection ability is also more restricted in subcortical regions for the 1.5T scanner compared to the 3T scanner (Nyberg et al., 2007). More importantly, the scanner confound risk making interpretations of group differences unreliable. Critically, the reported differences showed medium to large effect sizes on scanner-separated sub-analyses. Nevertheless, regions showing overlap between group differences and effects of scanner should be interpreted with caution. The relatively small PDMCI^+^ sample is a limitation, and affected the ability to generalize the findings to the general PD-population. Furthermore, our criteria for assessing MCI was not completely fulfilled (i.e., not two measures in each domain) which might have affected detection ability and also prevented sub-analyses of domain-specific MCI.

In conclusion, we report longitudinal BOLD-signal reductions in the right fusiform gyrus for PDMCI^+^ compared to PDMCI^−^. In addition, compared to PDMCI^−^, PDMCI^+^ showed persistent but non-changing under-recruitment across time in fronto-striatal circuitry in relation to deficits in working-memory updating. Taken together, the results are in keeping with the notion that the longitudinal development of cognitive impairment in PD reflect posterior cortical change rather than further fronto-striatal changes. Our population-based longitudinal approach thus adds further knowledge of how task-evoked brain responses relate to cognitive impairments in PD.

## Disclosure

Urban Ekman reports no disclosures. Johan Eriksson reports no disclosures. Lars Forsgren has received honoraria from Orion Pharma and GSK in the past 2 years. Magdalena E. Domellöf reports no disclosures. Eva Elgh reports no disclosures. Anders Lundquist reports no disclosures. Lars Nyberg reports no disclosures.

### Conflict of interest statement

The authors declare that the research was conducted in the absence of any commercial or financial relationships that could be construed as a potential conflict of interest.
