# Supplementary material for: Longitudinal changes in task-evoked brain responses in Parkinson's disease patients with and without mild cognitive impairment
Source: Front Neurosci. 2014 Jul 29;8:207. doi: 10.3389/fnins.2014.00207 (PMC4114183; doi:10.3389/fnins.2014.00207)
Supplement: Supplementary file 1 [file DataSheet1.DOCX]

**Supplementary material**

**Table 1. Confounding effects of scanner with the main effect of group analysis**

| **Brain region** | **Side** | **Peak (x, y, z)** | ***F*** | ***k*** |
| --- | --- | --- | --- | --- |
| **Patients without MCI > patients with MCI** | | | | |
| **Parietal** | | | | |
| Precuneus | R | 4, -68, 38 | 30.75 | 98 |
| Parietal/superior occipital | L | -20, -68, 40 | 27.74 | 98 |
| Precuneus | R | 20, -46, 14 | 25.48 | 158 |
| **Occipital** | | | | |
| Lateral occipital gyrus | L | -24, -96, 0 | 22.91 | 32 |
| Cuneus* | L | 2, -94, 20 | 13.33 | 50 |
| **Subcortical** | | | | |
| Putamen* | R | 24, 10, -4 | 14.67 | 40 |

Supplementary table 1 shows regions where the results from the main effect of scanner analysis partly overlapped with the reported group differences. p < 0.001. * = p < 0.005. However, quantifications of the responses for the two scanners separately showed consistent magnitude of BOLD-signal change on both scanners, with medium effect sizes (Cohen’s *d* >0.49) in left cuneus, left occipital cortex, and large effect sizes (Cohen’s *d* >0.80) in the additional areas. *F* = F-values. *k* = number of voxels.

**Note 1**

**Confounding effects of scanner with the group-by-time analysis**

Control analyses revealed an overlap between a group-by-scanner interaction effect and our reported group-by-time interaction effect in right cerebellum (18 voxels located in MNI-space x=10, y=-54, z=-32) and in left dorsolateral prefrontal cortex (109 voxels located in MNI-space x=-18, y=50, z=42; p < 0.005).

**Note 2**

**Confounding effects of scanner with the PPI analysis**

A control analysis revealed that the main effect of scanner partly overlapped with the reported effects of group in left precentral gyrus (40 voxels located in MNI-space x=-50, y=2, z=48), and in left superior intraparietal sulcus (37 voxels located in MNI-space x=-32, y=-62, z=54).
